# Using all Gene Families Vastly Expands Data Available for Phylogenomic Inference

**DOI:** 10.1093/molbev/msac112

**Published:** 2022-06-01

**Authors:** Megan L. Smith, Dan Vanderpool, Matthew W. Hahn

**Affiliations:** Department of Biology, Indiana University, Bloomington, IN, USA; Department of Computer Science, Indiana University, Bloomington, IN, USA; Department of Biology, Indiana University, Bloomington, IN, USA; Department of Computer Science, Indiana University, Bloomington, IN, USA; Department of Biology, Indiana University, Bloomington, IN, USA; Department of Computer Science, Indiana University, Bloomington, IN, USA

**Keywords:** phylogenetics, orthologs, paralogs, concatenation, coalescence

## Abstract

Traditionally, single-copy orthologs have been the gold standard in phylogenomics. Most phylogenomic studies identify putative single-copy orthologs using clustering approaches and retain families with a single sequence per species. This limits the amount of data available by excluding larger families. Recent advances have suggested several ways to include data from larger families. For instance, tree-based decomposition methods facilitate the extraction of orthologs from large families. Additionally, several methods for species tree inference are robust to the inclusion of paralogs and could use all of the data from larger families. Here, we explore the effects of using all families for phylogenetic inference by examining relationships among 26 primate species in detail and by analyzing five additional data sets. We compare single-copy families, orthologs extracted using tree-based decomposition approaches, and all families with all data. We explore several species tree inference methods, finding that identical trees are returned across nearly all subsets of the data and methods for primates. The relationships among Platyrrhini remain contentious; however, the species tree inference method matters more than the subset of data used. Using data from larger gene families drastically increases the number of genes available and leads to consistent estimates of branch lengths, nodal certainty and concordance, and inferences of introgression in primates. For the other data sets, topological inferences are consistent whether single-copy families or orthologs extracted using decomposition approaches are analyzed. Using larger gene families is a promising approach to include more data in phylogenomics without sacrificing accuracy, at least when high-quality genomes are available.

## Introduction

Advances in sequencing technology have led to the availability of more genomic data than ever before, and the promise of phylogenomics is the application of these data to infer species relationships (Scornavacca et al. 2020). Essential to the application of genomic data to phylogenetic inference is the identification of homologous genes, or genes that share a common ancestor. Homologous genes may share a common ancestor due to speciation (orthologs) or duplication (paralogs). Since the terms ortholog and paralog were coined (Fitch 1970), orthologs have been considered the appropriate genes for phylogenetic inference because they are related only through speciation events, and therefore, are thought to best reflect species relationships. Thus, identifying orthologs is a central part of most phylogenomic pipelines.

**Fig. 1.**
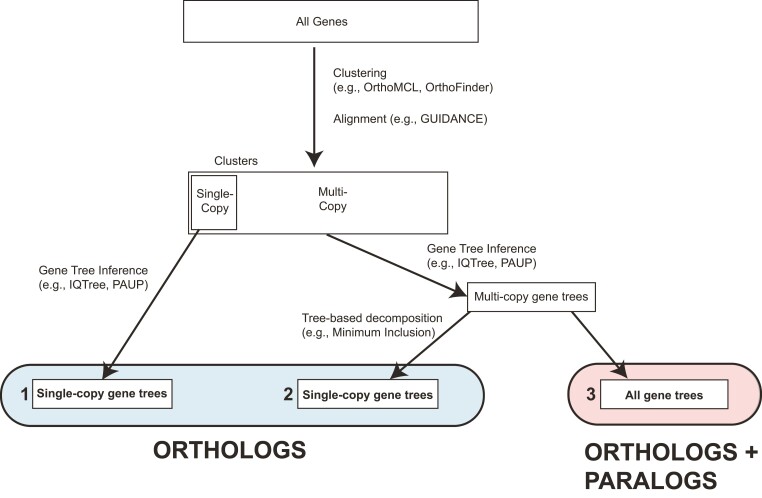
Conceptual overview of methods for inferring species trees from genomic data. We begin with All Genes, clustering them into gene families. We can then use single-copy ortholog clusters for inference (Data set 1), use tree-based decomposition approaches to extract orthologs from all clusters (Data set 2), or infer species trees from all clusters (i.e., from data sets including orthologs and paralogs; Data set 3).

Nearly all pipelines for extracting putative orthologs from genomic data begin with a clustering step (fig. 1). Clustering approaches aim to identify sets of homologous genes. While the details vary, these approaches generally begin with pairwise comparisons of all sequences across genomes, identify putative pairwise homologs, and then, use clustering approaches to attempt to group many sets of these genes together (reviewed in Altenhoff et al. 2019). The end-products of graph-based clustering approaches are clusters of orthologs and paralogs—i.e., gene families. Since most phylogenetic methods were designed for use with orthologs (and a single sequence per taxon), these groups must be further processed for downstream phylogenetic inference.

Three primary approaches have been used to process families for downstream inference (fig. 1; Step 1). The first and most common is to extract clusters with only a single copy in each species—these represent putative single-copy orthologs. Using single-copy families is generally seen as a conservative approach in phylogenomics, as these genes are likely to be orthologs; this choice also limits the amount of further downstream processing needed. However, the number of genes that are single copy in all sampled species decreases sharply as additional species are included in the analyses (Emms and Kelly 2018), limiting the usefulness of this approach in many phylogenetic contexts.

In lieu of relying only on single-copy clusters, tree-based decomposition approaches for orthology detection can be applied to extract orthologous genes from clusters that may have more than one copy in one or more species (fig. 1; Step 2). Tree-based decomposition approaches attempt to infer whether nodes in gene trees represent duplication or speciation events, followed by the extraction of orthologs based on these node labels (reviewed in Altenhoff et al. 2019). Early tree-based approaches relied on gene tree reconciliation to a known species tree (e.g., Goodman et al. 1979), limiting their utility in cases where the species tree is unknown or uncertain. However, recent approaches have relaxed these requirements. For example, the method LOFT relies on a species overlap approach to identify duplication nodes in gene trees (van der Heijden et al. 2007). Similarly, the software package Agalma (Dunn et al. 2013), the methods of Yang and Smith (2014), and the new method, DISCO (Willson et al. 2022), all extract subtrees without duplicates to generate sets of orthologs. While the exact implementations vary, in general, tree-based decomposition approaches aim to extract orthologous genes from families of any size. Tree-based approaches allow researchers to vastly increase the number of genes retained compared with using only the single-copy clusters. However, these approaches require that users construct gene trees and perform ortholog extraction for each gene family. Since gene trees must be constructed for all gene families, and some of these gene families may be rather large, these approaches can be substantially more computationally intensive than relying on single-copy clusters alone (fig. 1).

Finally, families containing both orthologs and paralogs could be used for phylogenetic inference. Although orthologs have traditionally been considered the appropriate genes for phylogenetics, methods for estimating phylogenies from data including paralogs were introduced more than 40 years ago (Goodman et al. 1979; reviewed in Smith and Hahn 2021). Recently, several popular methods for species tree estimation have been shown to be robust to the presence of paralogs (Hill et al. 2020; Legried et al. 2020; Markin and Eulenstein 2020; Yan et al. 2022). Of particular interest, quartet-based methods, such as ASTRAL (Zhang et al. 2018), should be robust to the inclusion of paralogs because the most common quartet is still expected to match the species tree even in the presence of gene duplication and loss. Given that all ortholog extraction methods may erroneously lead to the inclusion of paralogs, using methods that are robust to their inclusion is likely a good strategy—no matter the method employed to process the output of clustering methods.

Though there have been several empirical comparisons between ortholog-detection methods (e.g., Fernández et al. 2018; Kallal et al. 2018; Altenhoff et al. 2019), along with several simulation-based (e.g., Legried et al. 2020; Zhang et al. 2020; Morel et al. 2022; Yan et al. 2022) and empirical (e.g., Yan et al. 2022) studies evaluating the effects of paralog inclusion on phylogenetic inference, several questions remain. First, a comparison of inference on single-copy clusters to tree-based decomposition methods and methods that use all of the data (i.e., use orthologs and paralogs for phylogenetic inference) would shed light on the advantages of the three approaches. In addition, joint effects of data set, missing data requirements, and gene and species tree inference method on species tree topology will provide information on the importance of each. Finally, questions remain about the effects of the data set used on branch length estimates, measures of nodal support, and tests for introgression.

To address these questions, we focus our analysis on a recently published phylogenomic data set that includes 26 species of primates and 3 outgroups (Vanderpool et al. 2020). The data consist of whole genomes from all 29 species. In the original study, Vanderpool et al. restricted inference to 1,730 single-copy clusters present in 27 of the 29 studied species, a relatively small proportion of the >20,000 genes available from each species; the species tree was inferred using concatenated maximum likelihood (ML), concatenated maximum parsimony (MP), and quartet-based approaches applied to gene trees inferred using both ML and MP. The authors found robust relationships among all species except the Platyrrhini (“New World Monkeys”), for which inferences differed across species-tree and gene-tree inference methods. In this paper, we compare inferences from three major subsets of the data: single-copy families, orthologs extracted from larger families using tree-based decomposition approaches, and all families including all data (orthologs + paralogs). These data sets are then compared in three different phylogenetic applications. First, we compare the species trees inferred from these data sets using several methods, including concatenation-based and gene-tree based approaches. Second, we compare several measures of nodal support and nodal consistency, as well as branch length estimates across data sets. Finally, we perform tests of introgression and compare results across different data sets. In addition to analyzing the primate data set, we assembled data sets from five different groups (two fungi data sets, one plant data set, and two vertebrate data sets; Morel et al. 2022; Rasmussen and Kellis 2012), and compared species trees inferred from single-copy families, orthologs extracted from larger families using decomposition approaches, and all families for each. Our results suggest minimal effects of the subset of data used on downstream phylogenetic inference, while highlighting the fact that both tree-based decomposition approaches and approaches using both orthologs and paralogs greatly expand the amount of data available.

## Results

### Using All Gene Families Vastly Expands the Data Available for Phylogenetics in Primates

We compared three types of data sets produced by clustering approaches: single-copy clusters, orthologs extracted from all clusters using tree-based decomposition approaches, and all clusters (orthologs + paralogs) (fig. 1). For all data sets, we considered both a stringent missing data threshold (only those genes present in at least 27 of the 29 sampled species; MIN27) and a relaxed missing data threshold (only those genes present in at least 4 of the 29 sampled species; MIN4). Gene duplication and loss appear to have had a substantial impact on these data. For example, the 11,555 gene families sampled in 27 of 29 species included 428,129 gene copies (an average of 37 gene copies per gene family), and only a small fraction of these genes (1,820) were present in only a single copy in all sampled species. This suggests that most gene families studied here have experienced gene duplication and loss events during the evolutionary history of the primates. The first subset of the data considers only those clusters that included a single gene from each species (single-copy clusters; SCCs). While these genes are not guaranteed to be orthologs—due to the potential inclusion of pseudoorthologs (Doolittle and Brown 1994; Koonin 2005)—this is considered a safe approach and is often employed in phylogenomics. As expected, this data set included the fewest genes (table 1).

**Table 1.** Number of Primate Genes Trees and Gene Copies Included with Different Filtering Approaches.

Filter	MIN4		MIN27	
	Gene families	Gene copies	Gene families	Gene copies
**Single-copy clusters**	5,771	94,994	1,820	51,733
**Lineage-specific duplicates (LSD)**	13,627	297,831	7,693	219,441
**Two-species duplicates (TSD)**	14,931	332,718	8,719	248,759
**Maximum i** **nclusion**	27,880	331,990	4,849	137,733
**Maximum** **i** **nclusion (LSD)**	22,360	464,224	11,479	327,434
**Maximum** **i** **nclusion (TSD)**	21,793	473,000	12,046	343,652
**Monophyletic** **o** **utgroups**	9,724	200,503	4,805	136,749
**Monophyletic** **o** **utgroups (LSD)**	16,962	387,915	10,222	291,374
**Monophyletic** **o** **utgroups (TSD)**	17,104	390,584	10,254	292,257
**Subtree** **e** **xtraction**	20,562	470,465	12,198	347,994
**All** **p** **aralogs**	18,484	568,342	11,555	454,509
**One** **p** **aralogs**	18,484	428,129	11,555	330,115

LSD and TSD indicate when lineage-specific and both lineage-specific and two-species specific duplicates were trimmed; the SE method trims these automatically. The MIN4 data set required a minimum of 4 taxa (out of 29 total), while the MIN27 data set required a minimum of 27 taxa.

Tree-based decomposition approaches aim to extract orthologous genes from any cluster/family. We constructed gene trees for all clusters and then used several tree-based approaches to extract orthologous genes. First, we considered those clusters in which all duplications were specific to a single lineage and kept a single gene copy from this lineage. When duplications are restricted to a single lineage, choosing one of the copies as the ortholog cannot mislead phylogenetic inference regardless of which sequence is retained (see fig. 1d from Smith and Hahn 2021; supplementary fig. S1a, Supplementary Material online). This data set (“lineage-specific duplicates”; LSDs) included more than 4× as many genes as the SCC data set (table 1). Next, we further expanded our criteria to include those clusters with duplications specific to a pair of lineages (“two-species duplicates”; TSDs; supplementary fig. S1b, Supplementary Material online). Such duplications also cannot mislead topological inference, though picking a nonorthologous pair could lead to longer branches. It is straightforward to pick the most closely related pair of genes from the two species, which should not mislead either topological or branch length inferences; including these genes further expanded the data set compared with the LSD data set (table 1).

We considered two tree-based decomposition approaches from Yang and Smith (2014): maximum inclusion (MI) and monophyletic outgroups (MO). The MI approach takes a gene tree and iteratively extracts subtrees with the highest number of taxa without taxon duplication, until it cannot extract anymore subtrees with the minimum number of taxa. The MO approach considers only those gene trees with a monophyletic outgroup, roots the tree, and infers gene duplications from the root to the tips, pruning at nodes with duplications. These two approaches were each applied to three data sets: the original gene trees, the original gene trees trimmed to remove lineage-specific duplicates, and the original gene trees trimmed to remove both lineage-specific and two-species duplicates. We explored the effects of additional filtering and alternative parameters for the MI approach; as these changes had minimal effects, the results are presented in the supplementary Appendix A, Supplementary Material online. We also considered a new tree-based decomposition approach: subtree extraction (SE). In this approach, we midpoint-root gene trees, trimming away lineage-specific and two-species duplicates. We then extract subtrees that include a single representative from each taxon (i.e., subtrees with no duplicates) and keep those trees that meet minimum taxon-sampling thresholds (supplementary fig. S1c and d, Supplementary Material online).

All tree-based approaches further expanded the amount of data available (table 1). Since the SE and MI approaches are highly similar (neither requires an outgroup, and both aim to extract subtrees with no duplication events), we further examined the genes extracted using the two approaches. We compared the MI data set with two-species duplicates trimmed and a minimum of 27 taxa to the SE data set with a minimum of 27 taxa sampled (this method trims two-species duplicates internally). The number of trees extracted using the two approaches was very similar (12,046 vs. 12,198 genes in the MI and SE data sets, respectively). For the 12,046 trees in the MI data set, there was no analog in the SE data set for 2.4%, there was an identical tree in the SE data set for 92.7%, and there was a similar tree in the SE data set for 4.8% (median Robinson–Foulds distance of these trees = 2.0). Thus, the MI and SE approaches extract very similar subsets of trees from the original clusters.

Finally, we considered two approaches that made no attempt to remove paralogs from the data set. We considered one data set in which all orthologs and paralogs were included (“All Paralogs”). This data set was the most complete, as, even though it had fewer gene trees than some tree-based approaches, the gene trees from these tree-based approaches are subtrees extracted from this full data set. Therefore, this data set includes the most gene copies (table 1). This data set cannot be analyzed using concatenation methods because these approaches require an alignment that includes a single sequence for each species. To address this, and to evaluate the effects of stochastic sampling of paralogs, we also included a data set in which a single gene (without regard to whether it was an ortholog or paralog) was sampled at random from each species (“One Paralogs”).

**Fig. 2.**
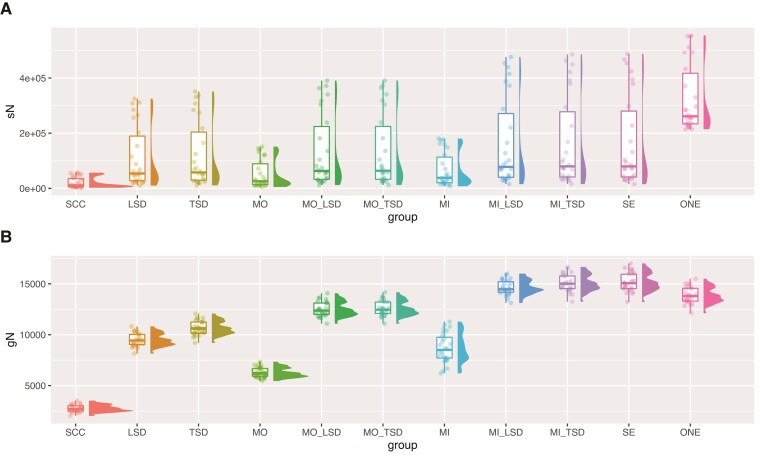
Numbers of informative genes and sites across data sets using the primate MIN27 data sets. (*A*) Distribution of the number of decisive sites (across branches) as calculated in IQ-Tree. Decisive sites are defined in Minh et al. (2020). (*B*) Distribution of the number of decisive gene trees (across branches) as calculated in IQ-Tree. Decisive gene trees are defined in Minh et al. (2020). SCC, single-copy clusters; LSD, lineage-specific duplicates; TSD, two-species duplicates; MO, monophyletic outgroup; MI, maximum inclusion; SE, subtree extraction; ONE, one paralogs.

**Fig. 3.**
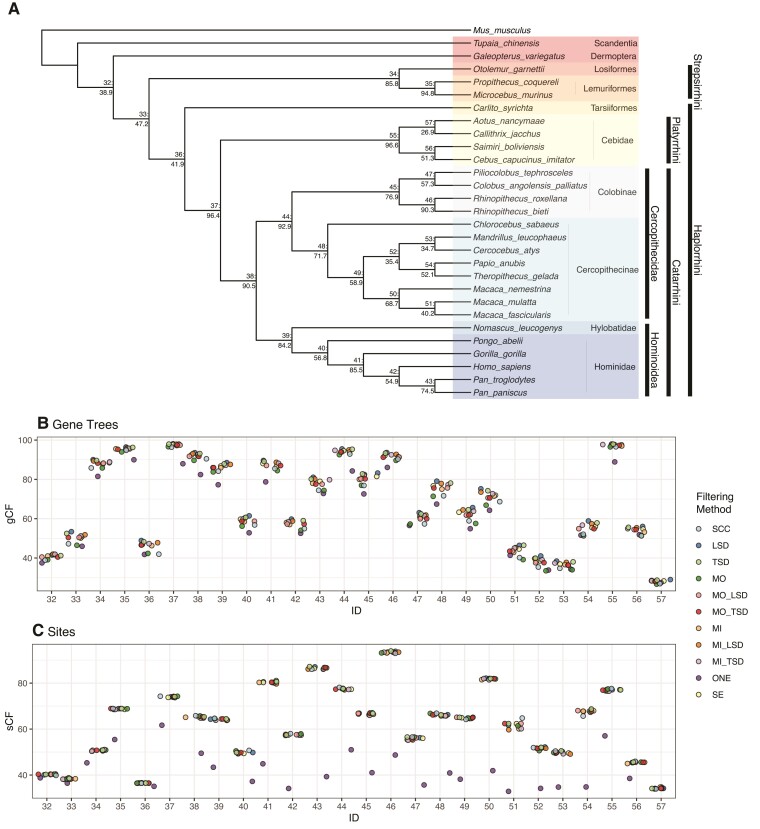
Gene (gCF) and site (sCF) concordance factors among primate data sets using ML gene trees (MIN27). (*A*) Primate phylogeny from ASTRAL-III using the ML gene trees (all input data sets give the same topology). Nodes show Node ID: gCF values from the SCC data set. (*B*) Distribution of gCF values across data sets. (*C*) Distribution of sCF values across data sets. Node IDs correspond to the numbers displayed on the tree in *A*. SCC, single-copy clusters; LSD, lineage-specific duplicates; TSD,  two-species duplicates; MO, monophyletic outgroup; MI, maximum inclusion; SE, subtree extraction; ONE, one paralogs.

In total, we considered 20 subsets of the data each with MIN4 and MIN27 taxon sampling. The number of gene families ranged from 1,820 to 27,900, and the number of gene copies ranged from 51,773 to 568,342 (table 1). Clearly, considering only SCCs drastically restricts the amount of data available, in terms of the number of gene trees (table 1), gene copies (table 1), decisive sites for each branch of the species tree (fig. 2A), and the gene trees informative about each branch of the species tree (fig. 2B). All other data sets are subsets of the All Paralogs data set, and thus, this data set is necessarily the most informative. Apart from the All Paralogs data set, including a randomly sampled paralog (One Paralogs) leads to the most decisive sites (fig. 2A), though they are not necessarily the most accurate sites (see below and fig. 3). MI and SE lead to the most informative gene trees (fig. 2B).

### Species tree inference is largely consistent across primate data sets

We inferred species trees using seven approaches: ASTRAL-III (Sayyari and Mirarab 2016; Zhang et al. 2018; Rabiee et al. 2019) on ML gene trees, ASTRAL-III on MP gene trees, ASTRID (Vachaspati and Warnow 2015) on ML gene trees, ASTRID on MP gene trees, concatenated ML inference in IQ-Tree (Nguyen et al. 2015), concatenated MP inference in PAUP* (Swofford 2001), SVDQuartets (Chifman and Kubatko 2014), ASTRAL-Pro (Zhang et al. 2020) on MP and ML gene trees, and ASTRAL-DISCO (Willson et al. 2022) on ML gene trees. ML gene trees were inferred in IQ-Tree, while MP gene trees were inferred in PAUP*. ASTRAL-III, ASTRID, concatenated ML, and concatenated MP were all developed with orthologs in mind, but ASTRAL-III has subsequently been demonstrated to be statistically consistent under models of gene duplication and loss when multiple copies are treated as multiple individuals or when a single copy per species is sampled (Hill et al. 2020; Legried et al. 2020; Markin and Eulenstein 2020). ASTRAL-Pro and ASTRAL-DISCO, on the other hand, were designed with paralogs in mind and were only applied to the All Paralogs data sets.

Across all nodes of the primate species tree, except for the relationships among the Platyrrhini (discussed below), an identical phylogeny was recovered across all data sets and species tree inference methods (fig. 3), with two exceptions. When concatenated MP or SVDQuartets was used to infer a species tree from the One Paralogs data set (MIN27), *Macaca fascicularis* was recovered as sister to *Macaca nemestrina* rather than *Macaca mulatta*, as in all other data sets and previous studies (e.g., Vanderpool et al. 2020). However, bootstrap support for this relationship was low (55%) in the SVDQuartets analysis. Additionally, when SVDQuartets was used to infer a species tree from the One Paralogs (MIN4) data set, *Mandrillus leucophaeus* was recovered as sister to a clade containing *Cercocebus atys, Papio anubis,* and *Theropithecus gelada*, rather than sister to *Cercocebus atys* as in other analyses and previous studies; bootstrap support for this relationship was also low (<50%).

Branch support values were also highly similar across filtering methods. Local posterior probabilities were 1.0 in ASTRAL-III for all data sets and nodes, except the contentious node in Platyrrhini. All local posterior probabilities were also 1.0 in ASTRAL-DISCO. All bootstrap support values in the concatenated ML analyses were 100, and all bootstrap support values were 100 in the concatenated MP analyses except for in the One Paralogs (MIN27) data set, which also had topological issues among macaques as mentioned above. Similarly, in all the SVDQuartets analyses, bootstrap values were 99 or 100, except among the Platyrrhini and in the One Paralogs data sets.

In addition to branch support values, we calculated measures of genealogical discordance: gene and site concordance factors (gCFs and sCFs; Minh et al. 2020). These analyses were carried out for all data sets except All Paralogs, because it is not possible to calculate these statistics for this data set in IQ-Tree, which requires a single sample per taxon. For all data sets except the One Paralogs data set, site and gene concordance factors were highly similar across data sets (fig. 3A–C). Concordance in the One Paralogs data set was consistently lower, as would be expected from the random sampling of homologs. In some cases, gene concordance factors were slightly lower for the SCC and MO data sets than for the other data sets (fig. 3B); this seems to be due to more genes that fall into the “paraphyly” category (i.e., genes for which at least one of the reference clades for a particular branch is not monophyletic), rather than for more genes supporting either of the two minor topologies. Gene and site concordance factors for the MIN4 data sets are shown in supplementary figure S2, Supplementary Material online.

### Resolution of the Platyrrhini Radiation Varies Across Species Tree and Gene Tree Inference Methods

**Fig. 4.**
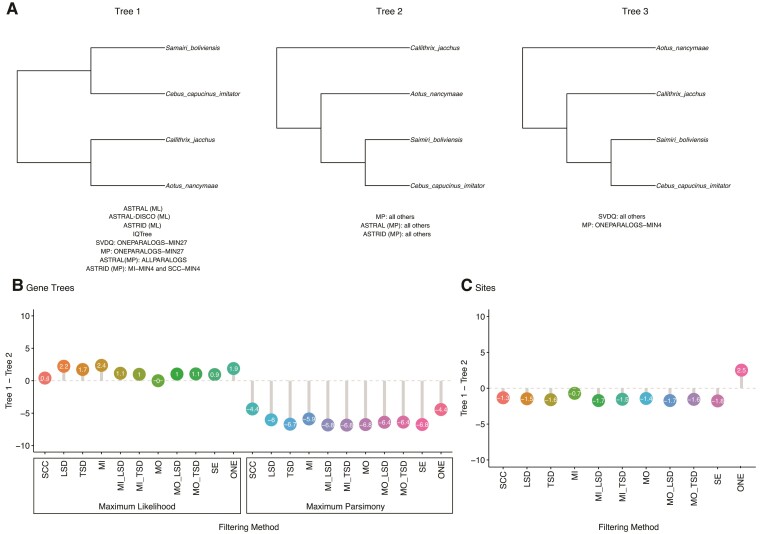
Alternative resolutions of Platyrrhini relationships. (*A*) The three most common tree topologies. Below each resolution, inference methods and filtering approaches that supported the topology are listed. (*B*) The percentage of gene trees supporting Tree 1 minus the percentage of gene trees supporting Tree 2 for ML and MP gene trees across data sets. (*C*) The percentage of sites supporting Tree 1 minus the percentage of sites supporting Tree 2 across data sets. SCC, single-copy clusters; LSD, lineage-specific duplicates; TSD, two-species duplicates; MO, monophyletic outgroup; MI, maximum inclusion; SE, subtree extraction; ONE, one paralogs. Results in *B* and *C* from MIN27 data sets.

As in Vanderpool et al. (2020), we found uncertainty around relationships among the Platyrrhini. Concatenated ML analyses and gene-tree based analyses that relied on gene trees inferred using ML preferred a symmetric tree, with *Saimiri boliviensis* and *Cebus capucinus imitator* as sister species and *Callithrix jacchus* and *Aotus nancymaae* as sister species (topology 1 in fig. 4A). However, concatenated MP and gene-tree based analyses that relied on gene trees inferred using MP preferred an asymmetric topology, with *S. boliviensis* and *C. c. imitator* sister and *A. nancymaae* sister to these two (topology 2 in fig. 4A). Finally, SVDQuartets preferred a third topology that placed *C. jacchus* sister to *S. boliviensis* and *C. c. imitator* (topology 3 in fig. 4A).

Gene and site concordance factors clarify these results. A slight majority of ML gene trees prefer topology 1 (fig. 4B), a majority of MP gene trees prefer topology 2 (fig. 4B), while slightly more sites support topology 2 than topology 1 (fig. 4C). While the results from SVDQuartets may seem counterintuitive at first, SVDQuartets relies on symmetry between the two minor topologies to infer the third topology as the correct topology. Since there are relatively equal numbers of sites supporting topologies 1 and 2, it is expected that SVDQuartets would prefer topology 3, even though fewer sites support this topology. Results for the MIN4 data set are similar and are shown in supplementary fig. S3, Supplementary Material online.

To further investigate the causes of disagreement among these taxa, we focused on the SCC data set with MIN27 filtering to compare ML and MP gene trees. For each gene, we recorded the ML and MP gene tree topology and the sCF with respect to the focal node, as well as various summary statistics about each locus (number of site patterns, number of parsimony informative sites, tree length, etc.). The percentage of sites supporting the best topology was highest when ML and MP gene trees agreed (supplementary fig. S6A and C, Supplementary Material online). Additionally, there was more variance in sCFs within a gene (i.e., the number of sites supporting each topology differed more) when ML and MP gene trees agreed (supplementary fig. S6A and B, Supplementary Material online). This suggests that for genes with similar numbers of sites supporting multiple topologies, ML and MP were more likely to infer conflicting gene trees. Notably, 17.6% of gene trees supported Tree 1 under both ML and MP inference, while 18.8% of gene trees supported Tree 2 under both ML and MP inference.

### Branch Length Estimates Are Largely Consistent Across Primate Data sets

**Fig. 5.**
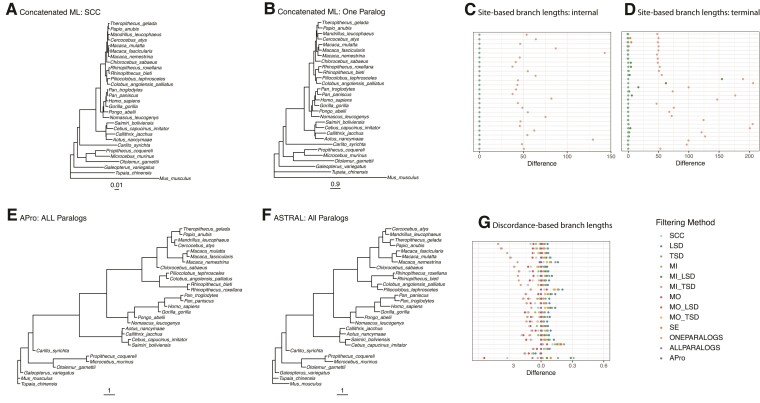
Branch lengths across primate data sets and species tree inference methods. Site-based branch lengths estimated using concatenated ML when (*A*) SCCs and (*B*) one randomly selected paralog per species are used for inference. Note the different scales in *A* and *B*. (*C*) Difference between site-based branch lengths for internal branches from the SCC data set and all the other data sets, normalized by SCC branch length. (*D*) Same as in *C*, but for terminal branches. Discordance-based branch lengths calculated on the All Paralogs data set when (*E*) ASTRAL-Pro and (*F*) ASTRAL-III are used for inference. Note that terminal branch lengths are arbitrary in these panels. (*G*) Difference between discordance-based branch lengths estimated with ASTRAL-Pro (APro) and all the other methods, normalized by APro branch length. Colors represent different filtering methods, and each row is a different branch. SCC, single-copy clusters; LSD, lineage-specific duplicates; TSD, two-species duplicates; MO,  monophyletic outgroup; MI, maximum inclusion; SE, subtree extraction; ONE, one paralogs. Results from MIN27 data sets.

We inferred branch lengths using two approaches. In general, our results suggest that all methods that extract orthologs perform similarly and should lead to reliable estimates of branch lengths. First, we estimated branch lengths in units of substitutions per site using concatenated ML (i.e., site-based branch lengths). We expect that the inclusion of paralogs will lead to an overestimation of the site-based branch lengths, since the divergence times of paralogs should pre-date the divergence times of orthologs. As expected, estimated site-based branch lengths for the One Paralogs data set are longer than those estimated for the SCC data set (fig. 5A and B). For all other MIN27 data sets, estimated site-based branch lengths were highly similar to those from the SCC data set (fig. 5C and D). However, there are some inconsistencies with the site-based branch lengths for terminal branches (fig. 5D), and all the site-based branch lengths are more variable for the MIN4 data sets (supplementary fig. S4, Supplementary Material online).

We also inferred discordance-based branch lengths in coalescent units using ASTRAL-III for the ML gene tree data sets. We expect that the inclusion of paralogs will lead to underestimated discordance-based branch lengths, because data sets with paralogs should have higher levels of discordance. As expected, the estimated discordance-based branch lengths from the All Paralogs and One Paralogs data sets using ASTRAL-III are shorter than those estimated from the All Paralogs data set using ASTRAL-Pro, a method that accounts for the extra discordance caused by the inclusion of paralogs (fig. 5E–G). In general, across all data sets except the two including paralogs (All and One), discordance-based branch lengths were highly similar to those estimated in ASTRAL-Pro (fig. 5G). However, there were some surprising results. Specifically, the SCC and MO data sets led to slightly shorter discordance-based branch length estimates than both ASTRAL-Pro and the data sets from other tree-based decomposition methods (fig. 5G). In addition, all discordance-based branch length estimates are relatively short, which could be explained by difficulties in estimating the lengths of longer branches with very little gene tree discordance (i.e., for which all [or most] genes support a single topology) in ASTRAL-III.

### Tests for Introgression Are Consistent Across Primate Data sets

**Fig. 6.**
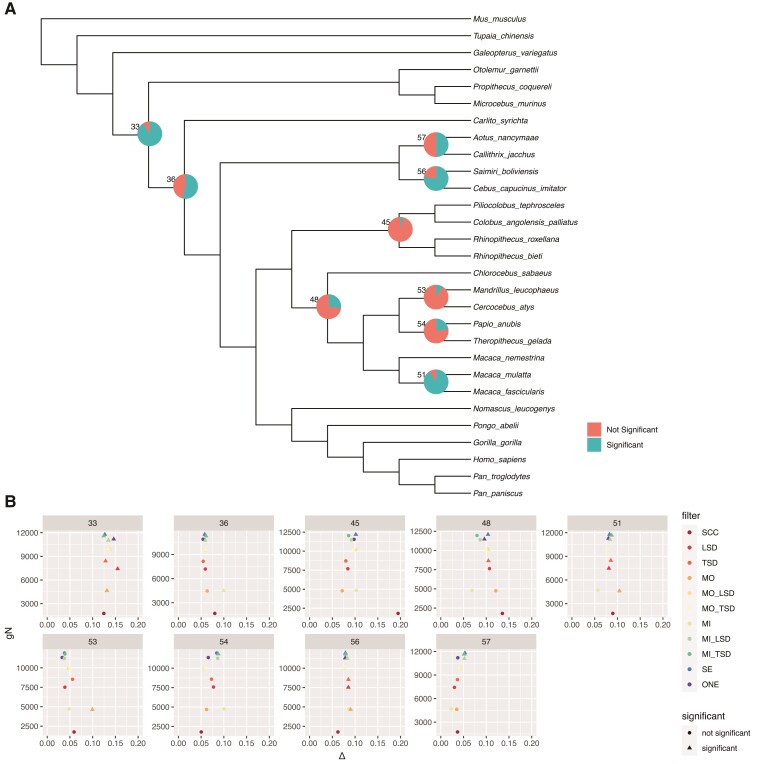
Results of introgression tests on primate MIN27 ML gene trees. (*A*) Pie charts are shown for branches with any significant introgression tests. Numbers are node numbers. (*B*) For all branches with some significant tests, we show the number of informative genes versus Δ. Observations are colored by filtering method, and shapes indicate whether a particular test was significant. SCC, single-copy clusters; LSD,  lineage-specific duplicates; TSD,  two-species duplicates; MO, monophyletic outgroup; MI, maximum inclusion; SE, subtree extraction; ONE, one paralogs.

To test for introgression, we looked for a deviation from the expected number of alternate gene tree topologies using the statistic Δ (Huson et al. 2005; Vanderpool et al. 2020). We used only the ML gene trees from each data set for this analysis. There was evidence of introgression across several branches of the primate phylogeny (fig. 6A), and values of Δ were similar across data sets (fig. 6B). Notably, there was evidence of introgression in a majority of tests at the contentious node in the Platyrrhini, which may explain difficulties inferring the species tree topology at this node. There was also evidence of introgression in the macaques, as found by Vanderpool et al. (2020). Deeper in the tree, results were more suspect, with tests on some data sets suggesting introgression while others did not (fig. 6B). The results of introgression tests were similar with less stringent missing data filters (supplementary fig. S5, Supplementary Material online).

### Inferred Species Trees Are Largely Consistent Across Additional Clades

We assembled data sets and inferred species trees for several other empirical data sets previously analyzed by Morel et al. (2022). We analyzed five data sets: a fungi data set including 16 species (fungi-16; Rasmussen and Kellis 2012), a fungi data set including 60 species (fungi-60; Huerta-Cepas et al. 2014), a vertebrate data set including 22 species (vertebrates-22; Huerta-Cepas et al. 2014), a vertebrate data set including 188 species (vertebrates-188; Zerbino et al. 2018), and a plant data set including 23 species (plants-23; Huerta-Cepas et al. 2014). These data sets varied widely in the number of gene copies (supplementary Appendix B, Supplementary Material online). The proportion of gene families that were single-copy ranged from ∼3% in the plants-23 data set to ∼67% in the fungi-16 data set. The data sets also varied in the number of gene families (supplementary Appendix B, Supplementary Material online), the number of taxa, and the depth of divergence. For each data set, we assembled seven subsets of gene families: SCCs, LSDs, TSDs, MI-extracted orthologs with two-species duplicates removed (MI-TSD), SE-extracted orthologs, All Paralogs, and One Paralogs. We then inferred species trees using ASTRAL-III, ASTRAL-Pro, ASTRID, concatenated ML, and concatenated MP. For three data sets, ASTRAL-III could not complete using the memory and wall-time available (up to 500 Gb and 94 h), so for these data sets, we used a modified version of FASTRAL (Dibaeinia et al. 2021). We omit results from other analyses that did not complete within 94 h of wall-time and 500 Gb of memory (supplementary Appendix B, Supplementary Material online).

**Fig. 7.**
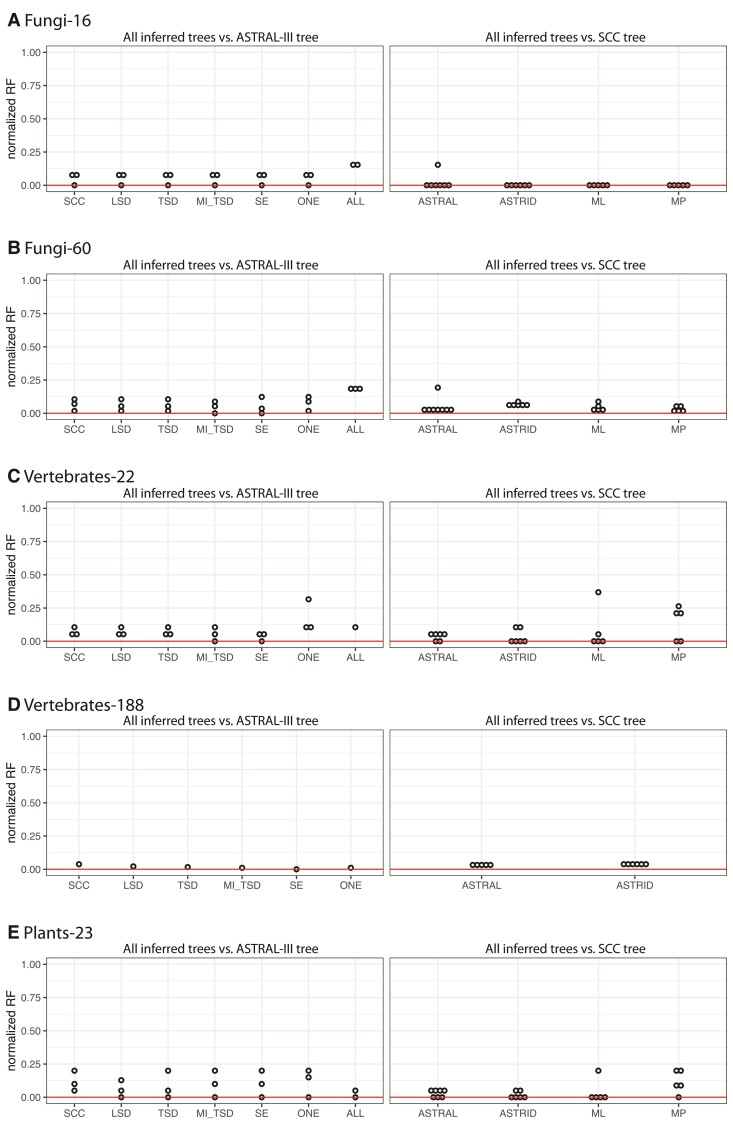
Results from analyzing five additional clades. On the left, we show the normalized Robinson-Foulds distances between trees inferred using different species tree inference methods (ASTRID, concatenated ML, concatenated MP, ASTRAL-Pro) and the tree inferred using ASTRAL-III for each data subset. On the right, we show the normalized Robinson-Foulds distances between trees inferred from different data subsets (SCC, LSD, TSD, MI-TSD, SE, ONE, ALL) and the SCC tree for each species tree inference method. (*A*) Fungi-16; (*B*) Fungi-60; (*C*) Vertebrates-22 (here, for the ‘All Paralogs’ data subset, the reference species tree on the left is the ASTRID tree, since ASTRAL-III did not complete); (*D*) Vertebrates-188; (*E*) Plants-23. SCC, single-copy clusters; LSD, lineage-specific duplicates; TSD, two-species duplicates; MO, monophyletic outgroup; MI, maximum inclusion; SE, subtree extraction; ONE, one paralogs.

In general, across any given inference method (e.g., all trees inferred with ASTRAL-III), species tree topologies were highly similar—whether we used SCCs or orthologs extracted from larger gene families (fig. 7; supplementary Appendix B, Supplementary Material online). The largest differences were between trees inferred using concatenated ML and concatenated MP on the one hand, and those inferred using the gene-tree based methods ASTRAL-III and ASTRID, on the other (Appendix B). Analyses of the One Paralogs subset using concatenated approaches resulted in highly different trees for the vertebrates-22 and plants-23 data sets (supplementary Appendix B, figs. B4 and B7, Supplementary Material online). In two cases, analyzing All Paralogs in ASTRAL-III resulted in different topologies as well. For the fungi-16 data set, the tree inferred in ASTRAL-III from All Paralogs differed from other trees at contentious nodes, but agreed with some previous studies (Rasmussen and Kellis 2012); nodal support values were also low at these nodes (supplementary Appendix B, fig. B1, Supplementary Material online). For the fungi-60 data set, the tree inferred from All Paralogs using ASTRAL-III was substantially different from other trees; our results suggest that this difference arose due to an issue when searching tree space in ASTRAL-III, rather than due to some inherent property of the data set (supplementary Appendix B, Supplementary Material online). Overall, our results highlight the robustness of topological inference to extracting genes from larger gene families, and in most cases, to using all data from all gene families.

## Discussion

Our results demonstrate that no matter the subset of the data used, the inferred species tree topology is largely stable; this was especially obvious in our analysis of primate genomes. Regardless of whether all families, families with only a single copy per species, or large families from which orthologs were extracted were used, the only disagreements between trees in the primate analyses were with respect to relationships among the Platyrrhini; in this case, the species tree inference method was a larger determinant of results than the particular data set (fig. 4). Despite the overall similarity among results, when a single gene was randomly sampled per species, results were unstable in two cases, suggesting—unsurprisingly—that such a sampling strategy is not ideal. Among additional data sets sampled from across the eukaryotes, results were also highly consistent whether SCCs or orthologs extracted from larger gene families were used for inference. While using all gene families resulted in consistent estimates of species tree topologies in most cases, analyzing these gene families with methods that were not designed for multicopy gene families (specifically, ASTRAL-III) resulted in an anomalous result in one case, likely due to issues appropriately searching tree space (supplementary Appendix B, Supplementary Material online). Based on the results presented here, when whole-genome sequence data are available, using all of the families output by clustering methods followed by the application of gene-tree decomposition methods can greatly expand the data available without sacrificing the accuracy of inference.

Several recent simulation studies have evaluated the impacts of gene duplication and loss on inferences of species tree topologies (Legried et al. 2020; Zhang et al. 2020; Morel et al. 2022; Yan et al. 2022). In studies considering the application of ASTRAL-III to multicopy gene families (i.e. using ASTRAL-multi), its performance has been surprisingly good, given that this method was not designed with duplication and loss in mind (Legried et al. 2020; Zhang et al. 2020; Yan et al. 2022). However, in some cases, this approach has been outperformed by methods that explicitly accommodate duplication and loss (Zhang et al. 2020; Willson et al. 2022), likely because these approaches use the information contained within gene duplication events, while limiting the effects of noise. ASTRAL-Pro (Zhang et al. 2020) includes an internal reconciliation step that labels speciation and duplication nodes, and is therefore operating similarly to gene tree decomposition approaches that try to identify such nodes in order to extract orthologs (although often not under any explicit model). In a comparison between ASTRAL-Pro and ASTRAL-DISCO (an approach that decomposes gene families prior to analyzing them in ASTRAL-III), ASTRAL-DISCO performed similarly to ASTRAL-Pro with lower computation times (Willson et al. 2022). Similarly, our analyses of six empirical data sets highlight the fact that tree-decomposition approaches perform similarly to ASTRAL-Pro when inferring species tree topologies. Taken together, these results suggest that decomposition is a promising approach for using a wider array of methods to infer species trees from large gene families.

Despite the stability of inference across most of the tree in the primate data set, there remains disagreement about relationships among the Platyrrhini, a notably contentious node (Perelman et al. 2011; Springer et al. 2012; Perez et al. 2013; Jameson Kiesling et al. 2015; Schrago and Seuánez 2019; Wang et al. 2019; Vanderpool et al. 2020). As in Vanderpool et al. (2020), we find that both concatenated ML and ASTRAL-III based on ML gene trees favor a symmetrical topology (tree 1 in fig. 4A). A bias toward the symmetrical 4-taxon tree is expected when using ML in the presence of recombination and when the time between speciation events is short (Kubatko and Degnan 2007; Roch and Steel 2015). Although the bias in ML under these conditions is often linked to concatenation methods, if the gene trees themselves are inaccurate due to the concatenation of multiple unique histories (e.g., among exons; Mendes et al. 2019), then the same bias in inferred trees can occur. Bias in the gene trees can then lead to bias in the methods that they are used as input to (e.g., ASTRAL-III). Note that this bias does not affect inferences under MP (Mendes and Hahn 2018). Furthermore, there are nearly equal numbers of trees supporting the two best-supported topologies in the primate data (fig. 4B), which suggests two things: first, choosing the best topology will be difficult no matter what method is used, as the evidence in favor of one topology over the other is minimal. Second, there is likely some introgression, since we would otherwise expect equal numbers of the two minor topologies. We do not see equal numbers of the two minor topologies, as confirmed by significant tests for introgression in this clade (fig. 6). Finally, a detailed comparison of SCC gene trees inferred by both ML and MP suggests that genes whose topologies disagreed across the two approaches did not support either topology as strongly as genes for which ML and MP agreed (supplementary fig. S6, Supplementary Material online). Of the gene trees that agreed across ML and MP inference, more supported Tree 2 than supported Tree 1 (fig. 4A). Thus, of the genes for which the methods agree, more support the asymmetric topology than the symmetric topology (as in Vanderpool et al. 2020).

We also compared branch length estimates and tests for introgression across data sets. Branch length estimates are largely consistent across data sets, with the exception of data sets that explicitly include paralogs, which led to biases in expected directions for both discordance-based and site-based branch lengths. Site-based branch lengths are very consistent across all data sets except the One Paralogs data set when stringent filters for missing data are applied. When paralogs are included, site-based branch lengths are overestimated, as expected (e.g., Siu-Ting et al. 2019). Discordance-based branch lengths (i.e., those estimated in ASTRAL) are underestimated for data sets including paralogs, because these data sets have higher levels of discordance. These methods accommodate increased discordance by positing a shorter time between speciation events. Otherwise, discordance-based branch lengths are largely similar across data sets, though the SCC and MO data sets appear to have slightly shorter estimated branch lengths than all other methods (fig. 5E). Given the consistency of results across tree-based decomposition methods, as well as ASTRAL-Pro, and the vastly larger number of gene trees used in these cases, we suggest that discordance-based branch lengths may actually be underestimated for the SCC and MO data sets. This result is consistent with lower gCFs in these data sets (fig. 3B) and suggests that branch lengths estimated from these data sets may be inaccurate because they include pseudoorthologs.

To our knowledge, this is the first evaluation of the effects of including more than just single-copy families on tests for introgression based on the asymmetry in minor topology frequencies. We expected that the inclusion of paralogs would not bias such tests, because under models that include duplication and loss, the two minor topologies should occur in equal frequencies (Smith and Hahn 2021, 2022). Our results largely confirm these expectations: although there is variation in whether or not tests are significant across data sets, estimates of Δ are very similar (fig. 6B). At some nodes, there is consistent evidence for introgression across data sets, suggesting a strong signal of asymmetry: for example, in the macaques and among the Platyrrhini. Deeper in the tree, there may be more gene tree error (e.g., due to long-branch attraction), since introgression is detected for some data sets and not for others (fig. 6B).

Phylogenetics based on whole-genome sequences almost always begins by identifying homologous genes via clustering. The clustering process operationally defines gene families, using clustering methods that range from very simple to very complex. While the single-copy clusters output by any one of these methods have most often been used in phylogenetics, there is nothing inherently more suitable about these clusters. First, SCCs may not be orthologs, due to the presence of pseudoorthologs—paralogs that are mistaken as orthologs due to differential patterns of gene duplication and loss (Doolittle and Brown 1994; Koonin 2005). In other words, having only a single representative sequence in each species does not guarantee that all the sampled genes are orthologs. Second, and more importantly, the size of clusters identified by clustering approaches is determined by parameters set by the user. For example, in OrthoMCL (Li 2003), the inflation parameter determines the size of output clusters: by changing this parameter, users can identify larger or smaller clusters. Because genes are related to all other genes via a long history of duplication and divergence (with a few exceptions; Knowles and McLysaght 2009; Zhao et al. 2014), there is no single level of similarity that uniquely identifies gene families (Demuth and Hahn 2009). However, users can choose the value of the inflation parameter that identifies more, smaller clusters, in order to find more single-copy clusters; this does not mean these genes do not have paralogs, only that more distant paralogs were not included at this clustering threshold. Many clustering methods aim to form groups of genes that descend from a single common ancestor in the studied taxa (e.g., Emms and Kelly 2015), though this does not ensure a lack of duplication events since the common ancestor. While tree-based decomposition approaches still rely on the clustering step to initially identify the homologs from which gene trees are built, their output is directly related to the definitions of orthologs and paralogs, and is more easily interpreted in a phylogenetic context. By applying these decomposition approaches to larger clusters, researchers can avoid arbitrary determinants of which clusters are single copy and can instead attempt to extract as many sets of orthologs as possible. Not only does this approach increase the amount of data available, but it also uses criteria more directly linked to the evolutionary history of gene families.

Our analyses included genomic data sets across vertebrates, plants, and fungi. While these data sets varied in the number of species, the depth of divergence, and the total number of available gene families, they are all relatively high-quality genomic data sets. Future works should investigate the effects of the inclusion of paralogs using data sets more prone to errors in homology inference and alignment. For example, when transcriptomic data are analyzed, not all homologs will necessarily be sequenced in all species, complicating the identification of orthologs and paralogs, even using tree-based decomposition approaches (Cheon et al. 2020). Target enrichment-based approaches (e.g., Faircloth et al. 2012; Weitemier et al. 2014) use probes to target-specific genomic regions and may inadvertently capture paralogous sequences. These data are generally limited to a moderate number of targeted orthologous regions, and the incidental inclusion of paralogs may have a much more pronounced effect, as there is far less signal available to overcome the noise associated with incorrect inferences of homology. Finally, inferences of homology may be more difficult when deeper phylogenetic problems are considered and in groups with frequent allo- and auto-polyploidy. These scenarios may challenge current phylogenomic methods in ways that the genomic data sets analyzed here do not, and should be carefully considered in future works.

In conclusion, our results suggest that methods for species tree inference are accurate across data sets, whether single-copy clusters or tree-based decomposition methods are used. For most subsets of the data and inference methods, using all clusters (i.e. paralogs and orthologs) also results in consistent inferences of species tree topologies. Our results highlight the benefits of using data from all gene families by showing that the amount of data used can be increased by an order of magnitude (table 1; fig. 2; supplementary Appendix B, Supplementary Material online). While even the smallest data set was sufficient for accurate species tree inference in the data sets analyzed here, that is not always the case (e.g., Emms and Kelly 2018; Thomas et al. 2020). In such cases, using only single-copy clusters may not be possible, and using data from larger gene families will be essential. Finally, more data facilitates inferences beyond species tree topology, including branch length estimates and the detection of introgression. Our results suggest that branch lengths estimated from single-copy clusters may be less consistent than those estimated using data from larger gene families in the primate data set (fig. 5), and adding gene families improves our ability to detect significant deviations from symmetric minor topology counts in tests for introgression (fig. 6). Our results are consistent across six empirical data sets that differ in the number of species, the number of gene families, the sizes of gene families, and the depth of divergence. While these data sets are not exhaustive, they suggest the potentially broad applicability of our findings, particularly with respect to the suitability of orthologs extracted from larger gene families for inferring species tree topologies.

## Materials and Methods

### Primate Data set and Alignment

The full sets of protein-encoding genes for 26 primates and 3 non-primates were obtained as in Vanderpool et al. (2020), and clusters were obtained as in that study. Briefly, an all-by-all BLASTP search (Altschul et al. 1990; Camacho et al. 2009) was executed, and the longest isoform of each protein-coding gene from each species was used. Then, the mcl algorithm (Van Dongen 2000) as implemented in FastOrtho (Wattam et al. 2014), with an inflation parameter of 5 was used to cluster the BLASTP output. CDSs for each cluster that included samples from at least four species were aligned, cleaned, and trimmed as in Vanderpool et al. (2020). Sequences were aligned by codon using GUIDANCE2 (Sela et al. 2015) with MAFFT v7.407 (Katoh and Standley 2013) with 60 bootstrap replicates. Sequence residues with GUIDANCE scores <0.93 were converted to gaps and sites with >50% gaps were removed using Trimalv1.4rev22 (Capella-Gutiérrez et al. 2009). GUIDANCE2 uses the command “mafft –localpair –maxiterate 1000 –nuc –quiet” when running MAFFT. Alignments shorter than 200 bp and that were invariant or contained no parsimony informative characters were removed from further analyses. Alignments that could not be aligned by codon were aligned by nucleotide, and subsequent steps were as with the codon-aligned data set. In total, 18,484 alignments were used in downstream analyses.

### Gene Tree Inference

We inferred gene trees from all alignments with at least four species (18,484 alignments) in IQ-TREE v2.0.6 (Nguyen et al. 2015) with nucleotide substitution models selected using ModelFinder (Kalyaanamoorthy et al. 2017) as implemented in IQ-TREE. The full IQ-TREE command used on each alignment was “iqtree2 -s *alignment name* -m MFP -c 1 -pre *alignment name*.” We also inferred gene trees from all 18,484 alignments using the MP criterion in PAUP* v 4.0a (Swofford 2001). We treated gaps as missing data, obtained a starting tree via random stepwise addition, held a single tree at each step, and used the TBR branch-swapping algorithm with a reconnection limit of 8. We kept a maximum of 1000 trees and did not collapse zero-length branches.

### Filtering

We considered three major groups of filtering methods:

1. Single-copy clusters: We considered a data set that consisted only of those clusters that included a single gene copy from each species.
2. Tree-based decomposition approaches: We considered several methods that involved trimming the branches of gene trees to extract orthologs. All custom branch-cutting operations were written in python3 and used the python package ete3 (Huerta-Cepas et al. 2016) to read, traverse, trim, and output gene trees and modified sequence alignments. We used postorder node traversal when traversing trees, and prior to custom trimming operations, we midpoint-rooted gene trees.
  - Lineage-specific duplicates: In this data set, we identified gene duplications that were specific to a single species. For such lineage-specific duplicates, we selected the sequence copy that was closest in length to the median length of sequences in the alignment, kept that copy, and trimmed the other copy or copies from both the alignment and the gene tree.
  - Two-species duplicates: To expand our data beyond LSDs, in addition to trimming lineage-specific duplicates, we identified gene duplications specific to a pair of species. For such duplicates, we selected the two sequence copies with the minimum branch distance separating them and trimmed the remaining copies from the tree and the alignment.
  - Maximum Inclusion: We applied the MI approach described in Yang and Smith (2014) to trim gene trees. We used the python script provided by Yang and Smith (2014; prune_paralogs_MI.py) and used as input one of three sets of gene trees: the original 18,484 gene trees, the original 18,484 gene trees with lineage-specific duplicates trimmed, and the original 18,484 gene trees with lineage-specific and two-species duplicates trimmed. For the MI approach, branches longer than a specified threshold are trimmed to remove potential pseudoorthologs; we used the following branch length cutoffs: 0.4 substitutions per site for the ML gene trees and 500 changes for MP trees. We explored additional cutoffs in the supplementary Appendix A, Supplementary Material online.
  - Monophyletic Outgroups: We also applied the MO approach described in Yang and Smith (2014) to trim gene trees. We used the python script provided by Yang and Smith (2014; prune_paralogs_MO.py) and used as input one of three sets of gene trees: the original 18,484 gene trees, the original 18,484 gene trees with lineage-specific duplicates trimmed, and the original 18,484 gene trees with lineage-specific and two-species duplicates trimmed.
  - Subtree extraction: Finally, we evaluated a new tree-based decomposition approach introduced here (SE). In this approach, we start by midpoint-rooting gene trees, followed by trimming lineage-specific and two-species duplicates. We then extract subtrees with a single representative from each taxon (i.e., subtrees with no duplicates) and keep those subtrees that meet minimum taxon-sampling thresholds.
3. Paralog methods: We considered two approaches that included paralogs in addition to orthologs. First, we included all genes (All Paralogs). Additionally, we randomly sampled a single gene (without regard to orthology) per species (One Paralogs).

For all data sets, we considered a stringent (minimum of 27 of 29 taxa) and relaxed (minimum of 4 of 29 taxa) missing data threshold.

### Species Tree Inference

We inferred species trees using seven methods. Three methods inferred species trees from concatenated data sets: MP, ML, and SVDQuartets. To infer an MP tree from the concatenated data sets, we used PAUP* v4.0a (build 168) (Swofford 2001). We set the criterion to parsimony, and used 500 bootstrap replicates to assess nodal support. For all other options, we used PAUP* defaults. To infer an ML tree from the concatenated data set, we used IQ-TREE v2.0.6 (Nguyen et al. 2015) with nucleotide substitution models selected using ModelFinder (Kalyaanamoorthy et al. 2017) as implemented in IQ-TREE. We used an edge-linked, proportional partition model (Chernomor et al. 2016) and 1000 ultrafast bootstrap replicates (Hoang et al. 2018). The full IQ-TREE command used on each alignment was “iqtree2 -s *alignment name* -p *partition file name* -c 1 -pre *alignment name -B 1000*.” For three alignments, IQ-Tree v2.0.6 failed to run, and, based on a suggestion from the developers, we reverted to IQ-Tree v.1.6.12 to infer the species trees for these alignments. For these three alignments, the full IQ-TREE command used was “iqtree -s *alignment name* -spp *partition file name* -pre *alignment name* -bb 1000 -nt 4.” Finally, to infer a species tree from the concatenated alignments using SVDQuartets, we used PAUP* v4.0a (build 168) (Swofford 2001). We evaluated all quartets and treated ambiguous sites as missing to infer the species tree topology using the command “svdq evalq = all bootstrap = no ambigs = missing loci = allchars;.” To assess nodal support, we evaluated 10,000 random quartets for each of the 100 bootstrap replicates. We used the multilocus bootstrapping option and again treated ambiguous sites as missing. The command used for bootstrapping in SVDQuartets was “svdq evalq=random nquartets=10000 bootstrap=multilocus loci=allchars nreps=100 nthreads=2 replace=yes treefile=*output file name* ambigs=missing;.”

In addition to the three concatenation-based methods, we inferred species trees using four gene-tree based methods. Prior to inferring species trees or estimating discordance (see below) from filtered gene trees, we collapsed all zero-length branches. For each gene tree, we did the following: first, we midpoint-rooted the gene tree. Then, we calculated sCFs using IQ-Tree v2.0.6 (Minh et al. 2020) for the alignment with the rooted gene tree as the reference tree. We used 100 randomly sampled quartets to compute the sCF, collapsing any nodes where sN == 0; in other words, any nodes for which no sites were informative.

We inferred a species tree using ASTRAL-III v5.7.3 (Sayyari and Mirarab 2016; Zhang et al. 2018; Rabiee et al. 2019). ASTRAL-III infers a species tree from a set of gene trees by extracting quartets and finding the species tree that maximizes the number of shared quartet trees. It has been demonstrated to be consistent under the multispecies coalescent (MSC) model (Mirarab et al. 2014) and under models of gene duplication and loss (Legried et al. 2020). Gene trees obtained using ML and MP, from all data sets described above, and with zero-length branches collapsed, were used as input to ASTRAL-III; local posterior probabilities were used to assess nodal support. In order to run ASTRAL-III on multicopy gene trees (i.e. the All Paralogs data set), we used the mapping file and treated each gene copy as a separate individual. Additionally, we inferred species trees using ASTRID v2.2.1 (Vachaspati and Warnow 2015), again using the filtered and zero-length collapsed ML and MP gene trees as input. ASTRID is a distance-based approach that estimates species trees using internode distances and is statistically consistent under the MSC model (Vachaspati and Warnow 2015). As in ASTRAL-III, for the All Paralogs data set, we treated gene copies from the same species as individuals using the mapping file. Finally, we inferred species trees from the All Paralogs data sets using ASTRAL-Pro (Zhang et al. 2020) and ASTRAL-DISCO (Willson et al. 2022). ASTRAL-Pro uses an internal rooting-and-tagging algorithm to label nodes as duplication or speciation nodes, and then infers quartets using only speciation nodes before finding the species tree that maximizes the number of shared quartet trees. ASTRAL-Pro has been shown to be statistically consistent under a model of gene duplication and loss, provided that rooting and tagging of nodes as speciation or duplication nodes is correct (Zhang et al. 2020). ASTRAL-DISCO decomposes multicopy gene trees into single-copy trees using the “rooting and tagging” algorithm from ASTRAL-Pro and then infers a species tree using ASTRAL-III.

### Assessing Discordance

To assess levels of discordance across data sets, we calculated gene and site concordance factors in IQ-Tree v2.0.6 (Minh et al. 2020). We used the tree shown in (fig. 3) as the reference tree, and to estimate sCFs, we used 1000 randomly sampled quartets. gCFs were estimated for filtered ML and MP gene trees after zero-length branches were collapsed. sCFs were estimated for the alignments that resulted from filtering the ML gene trees.

### Testing for Introgression

We used the approach used in Vanderpool et al. (2020) to test for introgression. Briefly, the introgression test assesses whether there is a deviation from the expected equal numbers of alternative tree topologies (under the MSC model without gene flow) using the statistic Δ (Huson et al. 2005), where

$$\Delta =\frac{\mathrm{Number}\;\mathrm{of}\;\mathrm{DF1}\;\mathrm{trees}-\mathrm{Number}\;\mathrm{of}\;\mathrm{DF2}\;\mathrm{trees}}{\mathrm{Number}\;\mathrm{of}\;\mathrm{DF1}\;\mathrm{trees}+\mathrm{Number}\;\mathrm{of}\;\mathrm{DF2}\;\mathrm{trees}}$$

DF1 represents the most common minor topology, and DF2 represents the least common minor topology. In the absence of introgression, Δ is expected to be equal to zero. To test whether the deviations from zero were significant, we followed the procedure of Vanderpool et al. (2020) and used 2,000 data sets generated by resampling gene trees with replacement, considering only those nodes where more than 5% of the trees were discordant. This distribution was used to calculate *Z*-scores and *P*-values for the observed Δ statistic, and for each filtered data set, we corrected for multiple comparisons using the Dunn–Sidák correction (Dunn 1959; Šidák 1967).

### Fungi, Vertebrate, and Plant Data sets

We downloaded the fungi-60, vertebrates-22, vertebrates-188, and plants-23 data sets from Morel et al. (2022). The fungi-60, vertebrates-22, and plants-23 data sets were extracted from the PhlomeDB database (Huerta-Cepas et al. 2014) by Morel et al. (2022). For these three data sets, amino acid matrices were used in concatenated analyses. We used gene trees from Morel et al. (2022) inferred from amino acid matrices using ParGenes (Morel et al. 2019) and RAxML-NG (Kozlov et al. 2019) for the fungi-60 and plants-23 data sets. For the vertebrates-22 data set, we followed Morel et al. (2022) in using the gene trees from the PhylomeDB database, which were reconstructed in PhyML v3.0 (Guindon and Gascuel 2003) from amino acid matrices. The vertebrates-188 data set was extracted from the Ensembl Compara database (Zerbino et al. 2018) by Morel et al. (2022). For this data set, nucleic acid matrices were used for concatenated analyses. We used gene trees from Morel et al. (2022) inferred from nucleic acid matrices using ParGenes (Morel et al. 2019) and RAxML-NG (Kozlov et al. 2019). We downloaded the fungi-16 data set (Rasmussen and Kellis 2012) from http://compbio.mit.edu/dlcoal/. For this data set, nucleic acid alignments were used for concatenated analyses, and we used gene trees from the original study inferred from nucleic acid matrices using PhyML (Guindon and Gascuel 2003). We removed two trees that had polytomies.

For each data set, we assembled seven subsets of gene families: SCCs, LSDs, TSDs, MI-extracted orthologs with two-species duplicates removed (MI-TSD), SE-extracted orthologs, All Paralogs, and One Paralogs. We inferred species trees using ASTRAL-III (Sayyari and Mirarab 2016; Zhang et al. 2018; Rabiee et al. 2019), ASTRID (Vachaspati and Warnow 2015), ASTRAL-Pro (Zhang et al. 2020), concatenated MP inference in PAUP* (Swofford 2001), and concatenated ML Inference in IQ-Tree (Nguyen et al. 2015). When ASTRAL-III could not complete within 94 h and 500 Gb, we ran FASTRAL (Dibaeinia et al. 2021). In order to run FASTRAL on data sets with missing data, we made slight changes to the FASTRAL source code by automating the construction of a custom map file for each run of ASTRID. We calculated distances between inferred trees using the python package ete3 (Huerta-Cepas et al. 2016).

## Supplementary Material

msac112_Supplementary_Data

## Data Availability

Scripts used for filtering gene trees are available on GitHub (github.com/meganlsmith/Primate_Paralogs). Primate alignments, gene trees, and species trees are available from FigShare (doi: 10.6084/m9.figshare.16653025).

## References

[msac112-B1] Altenhoff AM , Glover NM, Dessimoz C. 2019. Inferring orthology and paralogy. In: Anisimova M, editor. Evolutionary genomics: statistical and computational methods. New York (NY): Springer. p 149–175. 10.1007/978-1-4939-9074-0_5 31278664

[msac112-B2] Altschul SF , Gish W, Miller W, Myers EW, Lipman DJ. 1990. Basic local alignment search tool. J Mol Biol. 215:403–410. 2231712 10.1016/S0022-2836(05)80360-2

[msac112-B3] Camacho C , Coulouris G, Avagyan V, Ma N, Papadopoulos J, Bealer K, Madden TL. 2009. BLAST+: architecture and applications. BMC Bioinformatics 10:421. 20003500 10.1186/1471-2105-10-421 PMC2803857

[msac112-B4] Capella-Gutiérrez S , Silla-Martínez JM, Gabaldón T. 2009. trimAl: a tool for automated alignment trimming in large-scale phylogenetic analyses. Bioinformatics 25:1972–1973. 19505945 10.1093/bioinformatics/btp348 PMC2712344

[msac112-B5] Cheon S, Zhang J, Park C . 2020. Is phylotranscriptomics as reliable as phylogenomics? Mol Biol Evol. 37:3672–3683. 32658973 10.1093/molbev/msaa181 PMC7743905

[msac112-B6] Chernomor O , von Haeseler A, Minh BQ. 2016. Terrace aware data structure for phylogenomic inference from supermatrices. Syst Biol. 65:997–1008. 27121966 10.1093/sysbio/syw037 PMC5066062

[msac112-B7] Chifman J , Kubatko L. 2014. Quartet inference from SNP data under the coalescent model. Bioinformatics 30:3317–3324. 25104814 10.1093/bioinformatics/btu530 PMC4296144

[msac112-B8] Demuth JP , Hahn MW. 2009. The life and death of gene families. BioEssays 31:29–39. 19153999 10.1002/bies.080085

[msac112-B9] Dibaeinia P , Tabe-Bordbar S, Warnow T. 2021. FASTRAL: improving scalability of phylogenomic analysis. Bioinformatics 37:2317–2324. 10.1093/bioinformatics/btab093 PMC8388037 33576396

[msac112-B10] Doolittle WF , Brown JR. 1994. Tempo, mode, the progenote, and the universal root. Proc Natl Acad Sci U S A. 91:6721–6728. 8041689 10.1073/pnas.91.15.6721 PMC44275

[msac112-B11] Dunn OJ . 1959. Confidence intervals for the means of dependent, normally distributed variables. J Am Stat Assoc. 54:613–621.

[msac112-B12] Dunn CW , Howison M, Zapata F. 2013. Agalma: an automated phylogenomics workflow. BMC Bioinformatics 14:330. 24252138 10.1186/1471-2105-14-330 PMC3840672

[msac112-B13] Emms DM , Kelly S. 2015. OrthoFinder: solving fundamental biases in whole genome comparisons dramatically improves orthogroup inference accuracy. Genome Biol. 16:157. 26243257 10.1186/s13059-015-0721-2 PMC4531804

[msac112-B14] Emms DM , Kelly S. 2018. STAG: species tree inference from all genes. bioRxiv.

[msac112-B15] Faircloth BC , McCormack JE, Crawford NG, Harvey MG, Brumfield RT, Glenn TC. 2012. Ultraconserved elements anchor thousands of genetic markers spanning multiple evolutionary timescales. Syst Biol. 61:717–726. 22232343 10.1093/sysbio/sys004

[msac112-B16] Fernández R , Kallal RJ, Dimitrov D, Ballesteros JA, Arnedo MA, Giribet G, Hormiga G. 2018. Phylogenomics, diversification dynamics, and comparative transcriptomics across the spider tree of life. Curr Biol. 28:1489–1497.e5. 29706520 10.1016/j.cub.2018.03.064

[msac112-B17] Fitch WM . 1970. Distinguishing homologous from analogous proteins. Syst Zool. 19:99–113. 5449325

[msac112-B18] Goodman M , Czelusniak J, Moore GW, Romero-Herrera AE, Matsuda G. 1979. Fitting the gene lineage into its species lineage, a parsimony strategy illustrated by cladograms constructed from globin sequences. Syst Biol. 28:132–163.

[msac112-B19] Guindon S , Gascuel O. 2003. A simple, fast, and accurate algorithm to estimate large phylogenies by maximum likelihood. Syst Biol. 52:696–704. 14530136 10.1080/10635150390235520

[msac112-B20] Hill M , Legried B, Roch S. 2020. Species tree estimation under joint modeling of coalescence and duplication: sample complexity of quartet methods. arXiv:2007.06697.

[msac112-B21] Hoang DT , Chernomor O, von Haeseler A, Minh BQ, Vinh LS. 2018. UFBoot2: improving the ultrafast bootstrap approximation. Mol Biol Evol. 35:518–522. 29077904 10.1093/molbev/msx281 PMC5850222

[msac112-B22] Huerta-Cepas J , Capella-Gutiérrez S, Pryszcz L, Marcet-Houben M, Gabaldón T. 2014. Phylomedb v4: zooming into the plurality of evolutionary histories of a genome. Nucleic Acids Res 42(D):D897–D902. 24275491 10.1093/nar/gkt1177 PMC3964985

[msac112-B23] Huerta-Cepas J , Serra F, Bork P. 2016. ETE 3: reconstruction, analysis, and visualization of phylogenomic data. Mol Biol Evol. 33:1635–1638. 26921390 10.1093/molbev/msw046 PMC4868116

[msac112-B24] Huson DH , Klöpper T, Lockhart PJ, Steel MA. 2005. Reconstruction of reticulate networks from gene trees. In: Miyano S, Mesirov J, Kasif S, Istrail S, Pevzner PA, Waterman M, editors. Research in computational molecular biology. Vol. 3500. Lecture Notes in Computer Science. Berlin, Heidelberg: Springer. p 233–249. Available from: http://link.springer.com/10.1007/11415770_18

[msac112-B25] Jameson Kiesling NM , Yi SV, Xu K, Gianluca Sperone F, Wildman DE. 2015. The tempo and mode of New World monkey evolution and biogeography in the context of phylogenomic analysis. Mol Phylogenet Evol. 82:386–399. 24792088 10.1016/j.ympev.2014.03.027

[msac112-B26] Kallal RJ , Fernández R, Giribet G, Hormiga G. 2018. A phylotranscriptomic backbone of the orb-weaving spider family Araneidae (Arachnida. Araneae) supported by multiple methodological approaches. Mol Phylogenet Evol. 126:129–140. 29635025 10.1016/j.ympev.2018.04.007

[msac112-B27] Kalyaanamoorthy S , Minh BQ, Wong TKF, von Haeseler A, Jermiin LS. 2017. ModelFinder: fast model selection for accurate phylogenetic estimates. Nat Methods. 14:587–589. 28481363 10.1038/nmeth.4285 PMC5453245

[msac112-B28] Katoh K , Standley DM. 2013. MAFFT multiple sequence alignment software version 7: improvements in performance and usability. Mol Biol Evol. 30:772–780. 23329690 10.1093/molbev/mst010 PMC3603318

[msac112-B29] Knowles DG , McLysaght A. 2009. Recent de novo origin of human protein-coding genes. Genome Res. 19:1752–1759. 19726446 10.1101/gr.095026.109 PMC2765279

[msac112-B30] Koonin EV . 2005. Orthologs, paralogs, and evolutionary genomics. Annu Rev Genet. 39:309–338. 16285863 10.1146/annurev.genet.39.073003.114725

[msac112-B31] Kozlov AM, Darriba D, Flouri T, Morel B, Stamatakis A . 2019. RAxML-NG: a fast, scalable and user-friendly tool for maximum likelihood phylogenetic inference. Bioinformatics 35:4453–4455. 31070718 10.1093/bioinformatics/btz305 PMC6821337

[msac112-B32] Kubatko LS , Degnan JH. 2007. Inconsistency of phylogenetic estimates from concatenated data under coalescence. Syst Biol. 56:17–24. 17366134 10.1080/10635150601146041

[msac112-B33] Legried B , Molloy EK, Warnow T, Roch S. 2020. Polynomial-time statistical estimation of species trees under gene duplication and loss. J Comput Biol. 28:452–468. 33325781 10.1089/cmb.2020.0424

[msac112-B34] Li L . 2003. OrthoMCL: identification of ortholog groups for eukaryotic genomes. Genome Res. 13:2178–2189. 12952885 10.1101/gr.1224503 PMC403725

[msac112-B35] Markin A , Eulenstein O. 2020. Quartet-Based inference methods are statistically consistent under the unified duplication-loss-coalescence model. arXiv:2004.04299. 10.1093/bioinformatics/btab414 PMC9113308 34048529

[msac112-B36] Mendes FK , Hahn MW. 2018. Why concatenation fails near the anomaly zone. Syst Biol. 67:158–169. 28973673 10.1093/sysbio/syx063

[msac112-B37] Mendes FK , Livera AP, Hahn MW. 2019. The perils of intralocus recombination for inferences of molecular convergence. Philos Trans R Soc Lond [Biol]. 374:20180244. 10.1098/rstb.2018.0244 PMC6560264 31154973

[msac112-B38] Minh BQ , Hahn MW, Lanfear R. 2020. New methods to calculate concordance factors for phylogenomic datasets. Mol Biol Evol. 37:2727–2733. 32365179 10.1093/molbev/msaa106 PMC7475031

[msac112-B39] Mirarab S , Reaz R, Bayzid MdS, Zimmermann T, Swenson MS, Warnow T. 2014. ASTRAL: genome-scale coalescent-based species tree estimation. Bioinformatics 30:i541–i548. 25161245 10.1093/bioinformatics/btu462 PMC4147915

[msac112-B40] Morel B , Schade P, Lutteropp S, Williams TA, Szöllősi GJ, Stamatakis A. 2022. SpeciesRax: a tool for maximum likelihood species tree inference from gene family trees under duplication, transfer, and loss. Mol Biol Evol. 39:msab365. 35021210 10.1093/molbev/msab365 PMC8826479

[msac112-B41] Nguyen L-T , Schmidt HA, von Haeseler A, Minh BQ. 2015. IQ-TREE: a fast and effective stochastic algorithm for estimating maximum-likelihood phylogenies. Mol Biol Evol. 32:268–274. 25371430 10.1093/molbev/msu300 PMC4271533

[msac112-B42] Perelman P , Johnson WE, Roos C, Seuánez HN, Horvath JE, Moreira MAM, Kessing B, Pontius J, Roelke M, Rumpler Y, et al 2011. A molecular phylogeny of living primates. PLOS Genet. 7:e1001342. 21436896 10.1371/journal.pgen.1001342 PMC3060065

[msac112-B43] Perez SI , Tejedor MF, Novo NM, Aristide L. 2013. Divergence times and the evolutionary radiation of new world monkeys (Platyrrhini, Primates): an analysis of fossil and molecular data. PLoS One 8:e68029. 23826358 10.1371/journal.pone.0068029 PMC3694915

[msac112-B44] Rabiee M , Sayyari E, Mirarab S. 2019. Multi-allele species reconstruction using ASTRAL. Mol Phylogenet Evol. 130:286–296. 30393186 10.1016/j.ympev.2018.10.033

[msac112-B45] Rasmussen MD , Kellis M. 2012. Unified modeling of gene duplication, loss, and coalescence using a locus tree. Genome Res. 22:755–765. 22271778 10.1101/gr.123901.111 PMC3317157

[msac112-B46] Roch S , Steel M. 2015. Likelihood-based tree reconstruction on a concatenation of aligned sequence data sets can be statistically inconsistent. Theor Popul Biol. 100:56–62. 10.1016/j.tpb.2014.12.005 25545843

[msac112-B47] Sayyari E , Mirarab S. 2016. Fast coalescent-based computation of local branch support from quartet frequencies. Mol Biol Evol. 33:1654–1668. 27189547 10.1093/molbev/msw079 PMC4915361

[msac112-B48] Schrago CG , Seuánez HN. 2019. Large ancestral effective population size explains the difficult phylogenetic placement of owl monkeys. Am J Primatol. 81:e22955. 30779198 10.1002/ajp.22955

[msac112-B49] Scornavacca C , Delsuc F, Galtier N. 2020. Phylogenetics in the genomic era. Open access book. Available from https://hal.inria.fr/PGE/.

[msac112-B50] Sela I , Ashkenazy H, Katoh K, Pupko T. 2015. GUIDANCE2: accurate detection of unreliable alignment regions accounting for the uncertainty of multiple parameters. Nucleic Acids Res. 43:W7–W14. 25883146 10.1093/nar/gkv318 PMC4489236

[msac112-B51] Šidák Z . 1967. Rectangular confidence regions for the means of multivariate normal distributions. J Am Stat Assoc. 62:626–633.

[msac112-B52] Siu-Ting K , Torres-Sánchez M, San Mauro D, Wilcockson D, Wilkinson M, Pisani D, O’Connell MJ, Creevey CJ. 2019. Inadvertent paralog inclusion drives artifactual topologies and timetree estimates in phylogenomics. Mol Biol Evol. 36:1344–1356. 30903171 10.1093/molbev/msz067 PMC6526904

[msac112-B53] Smith ML , Hahn MW. 2021. New approaches for inferring phylogenies in the presence of paralogs. Trends Genet. 37:174–187. 32921510 10.1016/j.tig.2020.08.012

[msac112-B54] Smith ML , Hahn MW. 2022. The frequency and topology of pseudoorthologs. bioRxiv:2021.02.17.431499. 10.1093/sysbio/syab097 34951639

[msac112-B55] Springer MS , Meredith RW, Gatesy J, Emerling CA, Park J, Rabosky DL, Stadler T, Steiner C, Ryder OA, Janečka JE, et al 2012. Macroevolutionary dynamics and historical biogeography of primate diversification inferred from a species supermatrix. PLoS One 7:e49521. 23166696 10.1371/journal.pone.0049521 PMC3500307

[msac112-B56] Swofford DL. 2001. Paup*: Phylogenetic analysis using parsimony (and other methods) 4.0. B5.

[msac112-B57] Thomas GWC , Dohmen E, Hughes DST, Murali SC, Poelchau M, Glastad K, Anstead CA, Ayoub NA, Batterham P, Bellair M, et al 2020. Gene content evolution in the arthropods. Genome Biol 21:15. 31969194 10.1186/s13059-019-1925-7 PMC6977273

[msac112-B58] Vachaspati P , Warnow T. 2015. ASTRID: accurate species trees from internode distances. BMC Genomics 16:S3. 10.1186/1471-2164-16-S10-S3 PMC4602181 26449326

[msac112-B59] van der Heijden RT , Snel B, van Noort V, Huynen MA. 2007. Orthology prediction at scalable resolution by phylogenetic tree analysis. BMC Bioinformatics 8:83. 17346331 10.1186/1471-2105-8-83 PMC1838432

[msac112-B60] Vanderpool D , Minh BQ, Lanfear R, Hughes D, Murali S, Harris RA, Raveendran M, Muzny DM, Hibbins MS, Williamson RJ, et al 2020. Primate phylogenomics uncovers multiple rapid radiations and ancient interspecific introgression. PLOS Biol. 18:e3000954. 33270638 10.1371/journal.pbio.3000954 PMC7738166

[msac112-B61] Van Dongen SM. 2000. Graph clustering by flow simulation.

[msac112-B62] Wang X , Lim BK, Ting N, Hu J, Liang Y, Roos C, Yu L. 2019. Reconstructing the phylogeny of new world monkeys (platyrrhini): evidence from multiple non-coding loci. Curr Zool. 65:579–588. 31616489 10.1093/cz/zoy072 PMC6784508

[msac112-B63] Wattam AR , Abraham D, Dalay O, Disz TL, Driscoll T, Gabbard JL, Gillespie JJ, Gough R, Hix D, Kenyon R, et al 2014. PATRIC, the bacterial bioinformatics database and analysis resource. Nucleic Acids Res. 42:D581–D591. 24225323 10.1093/nar/gkt1099 PMC3965095

[msac112-B64] Weitemier K , Straub SCK, Cronn RC, Fishbein M, Schmickl R, McDonnell A, Liston A. 2014. Hyb-Seq: combining target enrichment and genome skimming for plant phylogenomics. Appl Plant Sci. 2:1400042. 10.3732/apps.1400042 PMC4162667 25225629

[msac112-B65] Willson J , Roddur MS, Liu B, Zaharias P, Warnow T. 2022. DISCO: species tree inference using multi-copy gene family tree decomposition. Syst Biol. 71:610–629. 34450658 10.1093/sysbio/syab070 PMC9016570

[msac112-B66] Yan Z , Smith ML, Du P, Hahn MW, Nakhleh L. 2022. Species tree inference on data with paralogs is accurate using methods intended to deal with incomplete lineage sorting. Syst Biol. 71:367–381. 34245291 10.1093/sysbio/syab056 PMC8978208

[msac112-B67] Yang Y , Smith SA. 2014. Orthology inference in nonmodel organisms using transcriptomes and low-coverage genomes: improving accuracy and matrix occupancy for phylogenomics. Mol Biol Evol. 31:3081–3092. 25158799 10.1093/molbev/msu245 PMC4209138

[msac112-B68] Zerbino DR , Achuthan P, Akanni W, Amode M, Barrell D, Bhai J, Billis K, Cummins C, Gall A, Girón CG, et al 2018. Ensembl 2018. Nucleic Acids Res. 46(D):D754–D761. 29155950 10.1093/nar/gkx1098 PMC5753206

[msac112-B69] Zhang C , Rabiee M, Sayyari E, Mirarab S. 2018. ASTRAL-III: polynomial time species tree reconstruction from partially resolved gene trees. BMC Bioinformatics 19:153. 29745866 10.1186/s12859-018-2129-y PMC5998893

[msac112-B70] Zhang C , Scornavacca C, Molloy EK, Mirarab S. 2020. ASTRAL-Pro: quartet-based species-tree inference despite paralogy. Mol Biol Evol. 37:3292–3307. 32886770 10.1093/molbev/msaa139 PMC7751180

[msac112-B71] Zhao L , Saelao P, Jones CD, Begun DJ. 2014. Origin and spread of de novo genes in *Drosophila melanogaster* populations. Science 343:769–772. 24457212 10.1126/science.1248286 PMC4391638

